# Determining the use of prophylactic antibiotics in breast cancer surgeries: a survey of practice

**DOI:** 10.1186/1471-2482-12-18

**Published:** 2012-08-31

**Authors:** Sergio A Acuna, Fernando A Angarita, Jaime Escallon, Mauricio Tawil, Lilian Torregrosa

**Affiliations:** 1 Department of Surgery, Hospital Universitario San Ignacio, Pontificia Universidad Javeriana, Bogotá, Colombia; 2 Department of Surgery, University of Toronto, Toronto, ON, Canada; 3 Department of Surgical Oncology, Princess Margaret Hospital, Toronto, ON, Canada; 4 Breast and Soft Tissue Clinic, Centro Javeriano de Oncología, Bogotá, Colombia; 5 Current address: Division of Experimental Therapeutics, Toronto General Research Institute, University Health Network, Toronto, ON, Canada; 6 Current address: Institute of Medical Science, University of Toronto, Toronto, ON, Canada

**Keywords:** Breast surgery, Surgical site infection, Prophylactic antibiotic

## Abstract

**Background:**

Prophylactic antibiotics (PAs) are beneficial to breast cancer patients undergoing surgery because they prevent surgical site infection (SSI), but limited information regarding their use has been published. This study aims to determine the use of PAs prior to breast cancer surgery amongst breast surgeons in Colombia.

**Methods:**

An online survey was distributed amongst the breast surgeon members of the Colombian Association of Mastology, the only breast surgery society of Colombia. The scope of the questions included demographics, clinical practice characteristics, PA prescription characteristics, and the use of PAs in common breast surgical procedures.

**Results:**

The survey was distributed amongst eighty-eight breast surgeons of whom forty-seven responded (response rate: 53.4%). Forty surgeons (85.1%) reported using PAs prior to surgery of which >60% used PAs during mastectomy, axillary lymph node dissection, and/or breast reconstruction. Surgeons reported they targeted the use of PAs in cases in which patients had any of the following SSI risk factors: diabetes mellitus, drains *in situ*, obesity, and neoadjuvant therapy. The distribution of the self-reported PA dosing regimens was as follows: single pre-operative fixed-dose (27.7%), single preoperative dose followed by a second dose if the surgery was prolonged (44.7%), single preoperative dose followed by one or more postoperative doses for >24 hours (10.6%), and single preoperative weight-adjusted dose (2.1%).

**Conclusion:**

Although this group of breast surgeons is aware of the importance of PAs in breast cancer surgery there is a discrepancy in how they use it, specifically with regards to prescription and timeliness of drug administration. Our findings call for targeted quality-improvement initiatives, such as standardized national guidelines, which can provide sufficient evidence for all stakeholders and therefore facilitate best practice medicine for breast cancer surgery.

## Background

Surgical site infection (SSI) of the breast is a source of postoperative complications that are not just limited to prolonged hospital stay and increased hospital costs, but also includes predisposing patients to additional medical interventions (e.g.: surgical debridement or abscess draining), poor aesthetic results, and psychological trauma [1-4]. More importantly SSI can delay adjuvant treatment [2], which can have a detrimental effect on a patient’s overall survival [5,6]. Certainly preventing SSIs in breast cancer patients is a necessary step for assuring high-quality surgical treatment.

In order to reduce SSI rates, the general recommendation is to give prophylactic antibiotics (PAs) one hour before starting surgery and to suspend them within the first twenty-four hours post-surgery [7]. Historically, using PAs in breast surgery was thought to be unnecessary [8] given that the breast is a peripheral soft tissue organ with no direct connection to any major body cavity or visceral structure [9] and that breast surgeries are typically classified as clean surgical procedures. Despite this premise, breast cancer surgery has been reported to have higher SSI rates (1.9 - 50%) than other clean surgical procedures [1,5,6,10-23]. Furthermore there are various studies in the indexed literature describing how PAs can decrease SSI rates in this surgical procedure [7]. Even with this evidence there is still no general consensus in the literature [7] therefore, evaluating current practice patterns amongst breast surgeons can be used to build a framework to establish best practice guidelines at a national level. This study aims to determine the use of PAs prior to breast cancer surgery amongst specialists in Colombia.

## Methods

Approval from the research ethics board at Hospital Universitario San Ignacio in Bogota, Colombia was obtained prior to starting this study. An online survey was developed in Spanish using a commercial, Internet-based service (Encuesta Fácil, S.L.). The survey consisted of eleven questions (Table 1) that included the following topics: demographics, clinical practice characteristics, and the use of PA in common breast surgical procedures. The questions were developed in Spanish, but for the purpose of this publication they are provided in English.

**Table 1:** Translated survey questions

**Number**	**Question**	**Possible answer(s)**
1	In what city do you practice?	*Open answer.*
2	What is your specialty?	*Choose one of the following:* breast surgery, surgical oncology, general surgery, gynecology/obstetrics, or plastic surgery.
		
3	How many years of practice do you have in breast surgery?	*Open answer.*
4	What type of practice do you have?	*Choose one of the following:* Private or private/academic
5	What percentage of your cases corresponds to breast surgery?	*Choose one of the following:* <25%, 25– 49%, 50– 75%, or >75%.
6	What is your monthly breast surgery case load?	*Choose one of the following:*<5 cases/month, 5 – 15 cases/month, 16 – 25 cases/month, or >25 cases/month.
		
7	Indicate from the following list of breast surgical procedures in which cases you administer prophylactic antibiotic:	*Select as many as are appropriate*: breast conserving surgery, wire localized excision, mastectomy, axillary lymph node dissection, sentinel lymph node biopsy, reconstruction with flap, reconstruction with implant, terminal conduct excision, and benign lesion excision.
8	Do you use prophylactic antibiotic in all your breast surgeries?	*Choose one of the following*: yes or no.
9	What prophylactic antibiotic do you use?	*Open answer.*
10	If you use prophylactic antibiotic, how do you administer it?	*Choose one of the following*: single pre-operative fixed-dose, single preoperative fixed dose followed by a second fixed dose if the surgery is prolonged, single preoperative fixed dose followed by one or more postoperative fixed doses for >24 hours, or single preoperative weight-adjusted dose.
11	If you do not administer routine prophylactic antibiotic, in what cases do you use it?	*Select as many as are appropriate*: older age, obesity, cancer, smoking, diabetes mellitus, active skin disease, neoadjuvant therapy, use of drains *in situ*, and surgical re-intervention.

Before distributing the survey it was validated for internal congruency and user friendliness by conducting a small pilot study with five breast surgeons, which were not included in the final data analysis of this study. The survey was distributed between November 2009 and March 2010 exclusively amongst breast surgeons in Colombia. A list of names and contact information (electronic mail) was obtained from the national breast surgery society's database [Asociación Colombiana de MastologÃa (English: Colombian Association of Mastology)].

Respondents were allowed to modify their answers to previous questions, but were unable to edit or resubmit the survey once it was completed. Answers were automatically kept anonymous. A reminder was sent out to those who had not responded two and four months after the survey was originally distributed. No rewards or incentives were given for completion of this survey. Descriptive statistical analysis was carried out using means, medians, standard deviations, and ranges using SPSS 19.0 (IBM, Chicago, IL).

## Results

Surveys were distributed amongst all the eighty-eight breast surgeon members of the Colombian Association of Masology of which forty-seven completed the whole survey (response rate: 53.4%). Demographic information is described in Table 2. All of the surgeons reported that they lived in major urban areas in Colombia. Furthermore, 76.6% of the respondents practiced in academic hospital settings. Approximately forty percent of surgeons reported that they performed between 5 and 15 breast surgeries per month.

**Table 2:** Demographic data of survey respondents

**Characteristic**	
**Type of specialty, N (%)**	
Breast surgery	23 (48.9)
Surgical oncology	5 (10.6)
General surgery	6 (12.8)
Plastic surgery	13 (27.7)
Years of practice in breast surgery, mean ± SD (range)	13.2 ± 9.9 (1–45)
**Type of practice, N (%)**	
Private	11 (23.4)
Private/academic	36 (76.6)
**Percentage of cases corresponding to breast surgery, N (%)**	
<25	9 (19.1)
25 – 49	9 (19.1)
50 – 75	4 (8.5)
>75	25 (53.2)
**Volume of breast surgeries per month, N (%)**	
<5 cases	2 (4.3)
5 – 15 cases	19 (40.4)
16 – 25 cases	9 (19.1)
>25 cases	17 (36.2)

Only forty surgeons (85.1%) reported that they administered a PA before breast surgeries. The self-reported PA dosing regimens used by this group of surgeons was as follows: single pre-operative fixed-dose (27.7%), single preoperative fixed dose followed by a second fixed dose if the surgery was prolonged (44.7%), single preoperative fixed dose followed by one or more postoperative fixed doses for >24 hours (10.6%), and single preoperative weight-adjusted dose (2.1%). The antibiotic of choice was unanimously cefazolin.

**Figure 1:**
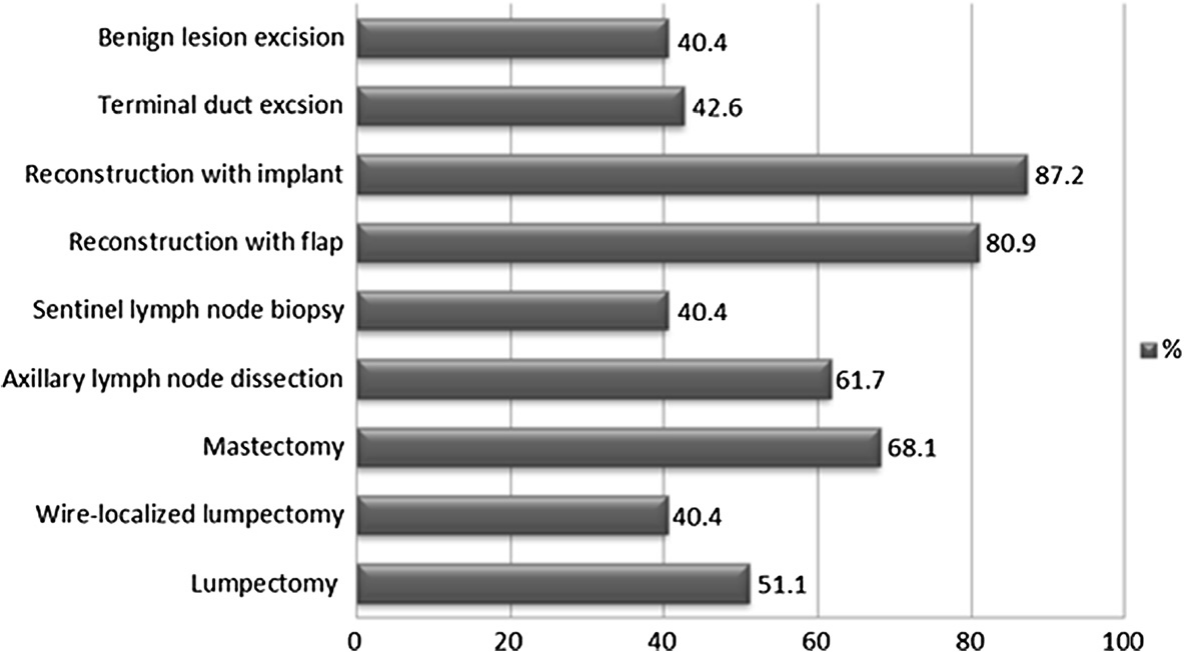
**Common breast surgical procedures in which breast surgeons use prophylactic antibiotics.** The most common surgical procedures in which surgeons reported they used PAs were breast reconstruction with implant or flap, mastectomy, and axillary lymph node dissection.

**Figure 2:**
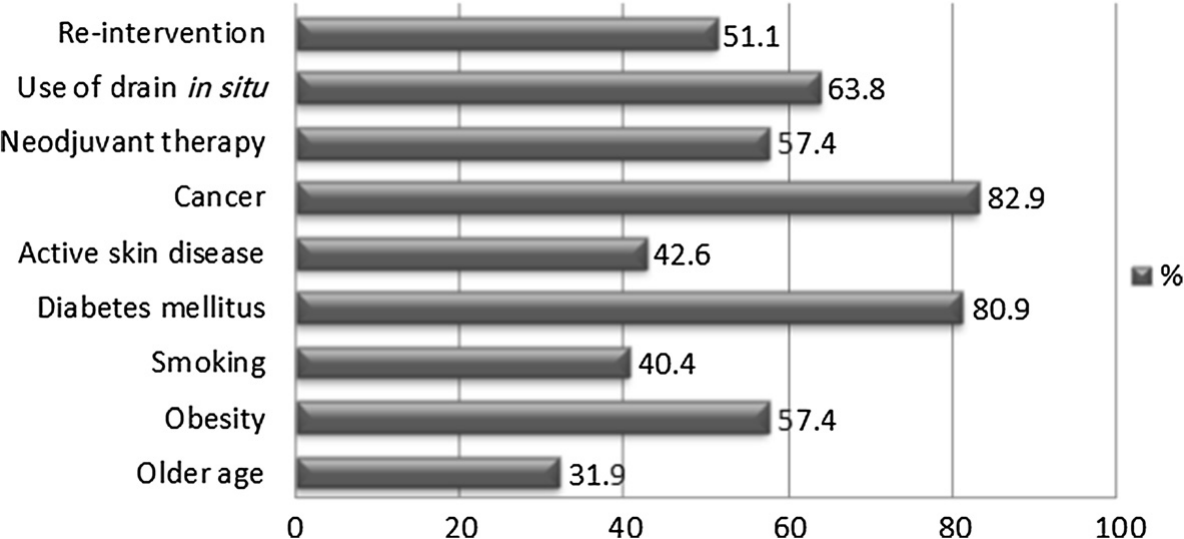
**Breast cancer patient characteristics reported to be taken into consideration for targeted prophylactic antibiotic use in breast cancer surgery.** Surgeons who reported using targeted prophylaxis considered the following patient characteristics to be the most important ones when considering who should receive PAs: cancer, diabetes mellitus, use of drain *in situ*, neoadjuvant therapy, and surgical re-intervention.

Surgeons who reported using PAs were then asked about the specific breast surgical procedure in which they used them. The distribution of these surgical procedures is shown in Figure 1. The most common procedures in which PAs were administered included breast reconstruction with implant (87.2%) or flap (80.9%), mastectomy (68.1%), and axillary lymph node dissection (61.7%). Nineteen surgeons (40.4%) reported that they used PAs routinely in all their breast surgeries and the remaining twenty-eight surgeons (59.6%) stated that they used targeted prophylaxis while taking into consideration various, specific patient characteristics. The distribution of these patient characteristics is outlined in Figure 2.

## Discussion

Appropriately selected and timely prophylactic antimicrobial agents are proven to decrease SSI rates in breast cancer patients undergoing surgical treatment [22-24]. Surgeons may hesitate to follow this recommendation because uncontrolled and injudicious use of PAs may lead to antibiotic resistance [25], adverse effects (e.g.: *Clostridium difficile* infection) [26], and increase medical costs [27] because they decrease SSI symptoms until after the patient has been discharged [28]. Nevertheless, the benefits related to this measure outweigh the sporadic number of complications.

Our study shows that the majority of breast surgeons that responded to this survey use some type of PA in breast cancer patients before surgery whether it is administered routinely in all patients or selectively when dealing with specific high-risk variables associated with SSI. Nonetheless, among those surgeons that reported using PAs the practice pattern is heterogeneous. In the literature there is evidence that this disparity is also common amongst surgeons from other countries. For example, a British survey reported that up to 33% of surgeons who performed wide local excisions, mastectomies and sentinel lymph node biopsy used PAs [29]. Another study carried out in Spain reported that 52% of hospitals used PAs in breast surgery [30] although a more recent multi-centric Spanish study revealed that the rate of use of PAs was much higher (97.81%) [31]. These results must be analyzed cautiously because breast cancer surgeries are not always carried out by breast surgeons or other sub-specialized surgeons so there may be a bias in the way the information is gathered.

Studies have evaluated the impact of PAs, but have showed mixed results. Two studies reported a reduction in SSI rates that ranged from 33 to 88% after using cefotaxime and azithromycin [1,22]. Other researchers have not found any significant reduction in SSI rates [23,28,32]. Nonetheless, a Cochrane review concluded that using pre-operative antibiotics significantly reduces the risk of SSI (pooled risk ratio 0.71, 95% confidence interval 0.53-0.94) in patients undergoing surgery for breast cancer when compared with placebo or no treatment [7]. This type of prophylactic intervention is reported to potentially benefit high-risk patients especially when they have any of the following risk factors: neoadjuvant chemotherapy, immediate breast reconstruction, blood transfusion, obesity, and smoking [24].

In our study 80% of surgeons reported that they used PAs in patients undergoing breast reconstruction. In a survey of the members of the American Society of Plastic Surgeons the use of PAs was slightly higher (>90%) [33]. The authors stated that plastic surgeons use PAs in patients undergoing any type of cosmetic or reconstructive breast surgery because a higher rate of SSI would exist if they were not used and also because these types of surgeries *per se* increase the risk of SSI as they have a longer length of duration and use foreign bodies (e.g.: implants). This concept certainly goes along with the recommendation made by the Hospital Infection Control Practices Advisory Committee of the U.S. Centers for Disease Control and Prevention in which clean procedures require antibiotic prophylaxis when implanting foreign material and in any case where an SSI may pose a catastrophic risk [34].

In addition to the standard SSI risk factors inherent to any patient (e.g.: obesity, history of smoking, diabetes, etc.) [34], breast cancer surgery patients have additional, specific risk factors (e.g.: neoadjuvant chemotherapy, re-operations, use of foreign bodies such as implants and drains *in situ*, and post-operative seroma) [14] that increase their susceptibility to post-operative infections. As a result of this, breast cancer patients exceed the 1.5% SSI rate suggested for elective clean surgery [35,36]. Accordingly, at least 40% of the breast surgeons within our study reported that they administered PAs specifically when their patients had any of these SSI risk factors.

The details of drug choices amongst surveyed surgeons are in line with current recommendations. PAs are typically directed against gram-positive bacteria that comprise normal skin flora (staphylococci and streptococci). Ng *et al.* reported that British surgeons tend to use amoxicillin-clavulonic acid more often than cephalosporins [29]; however at many institutions cefazolin is preferred. For example, Codina *et al.* reported that the majority (36%) of hospitals in Spain prefer cefazolin [30]. Studies evaluating the effectiveness of cephalosporins to reduce breast surgery SSI have had mixed results [20,32,37]. In the past, most breast surgery SSIs were caused by staphylococci and streptococci [5,12,13], but recent data suggests that there are significant rates (30–66.2%) of non-staphylococcal infections [35-39]. Additionally, 63% of the staphylococcal isolates have been documented to be resistant to at least one antibiotic [39]. Breast surgeons should be aware of this fact and monitor patients with complicated wounds that do not respond to standardized treatments. In the future there may be a need to change the PAs we are currently using.

Our survey brings to light a couple of issues regarding standardized prescription practices that require improvement in the clinical practice of breast surgeons in Colombia. In this survey only 2.1% of the breast surgeons answered that they actually weight-adjust their preoperative dosing. Although cefazolin is an antibiotic with prolonged half-life, its ability to prevent SSI is significantly affected by sub-optimal dosing therefore in order to assure optimal drug concentrations appropriate weight adjusted dosing and re-dosing is mandatory. On another note, the self-reported timeliness of antibiotic administration is compliant with current recommendations [40] in 89.4% of the breast surgeons we surveyed. Despite the fact that the majority of breast surgeons in this cohort understand the essential role of when and how long PAs should be administered to actually prevent SSIs, there are a significant number of surgeons that reported they extended the use of PAs beyond the first 24 hours post-surgery exclusively with the intention to reduce the risk of SSI. A similar practice pattern has been reported in Spain in which 9% of surveyed hospitals self-reported that their surgeons prolonged the use of PAs for over 24 hours when performing breast surgeries [30]. Randomized studies have shown that administering PAs for only 24 hours is enough to prevent SSIs and that prolonging its use does not provide any additional benefit [41], but instead increases the risk of generating resistant bacterial strains [42], nosocomial infection [42], diarrhea [26], higher health-care costs [27], and increased work load for health-care staff [43].

## Conclusion

SSIs, which are commonly associated with breast cancer surgery, require extreme attention amongst breast surgeons because they can take several specific actions to decrease the incidence. One of the preventative measures is appropriately administering PAs. Our study shows that although our cohort of breast surgeons is aware of their importance, variation exists in terms of how PAs are prescribed, specifically with regards to dosing and timeliness. Findings such as the ones described in this study call for the development of targeted quality-improvement initiatives, such as guidelines, that can ensure best practice medicine for breast cancer surgery in Colombia.

## Competing interests

The authors declare that they have no competing interests.

## Authors’ contributions

FAA conceived the study, carried out the survey, participated in the statistical and data analysis and the design of the study, and helped to draft the manuscript. SAA carried out the survey, participated in the data analysis and the design of the study, and helped to draft the manuscript. JE participated in data analysis and helped to draft the manuscript. MT participated in the design of the study and data analysis and helped to draft the manuscript. LT conceived the study, carried out the survey, participated in its design and was the main coordinator. All authors read and approved the final manuscript.

## Pre-publication history

The pre-publication history for this paper can be accessed here:

http://www.biomedcentral.com/1471-2482/12/18/prepub
